# Impact of air pollution and asthma on school attendance and educational attainment: a scoping review

**DOI:** 10.1136/bmjresp-2025-003527

**Published:** 2025-12-07

**Authors:** Cedric Burden, Zakariah Gassasse, Mohammed Alsallakh, Jennifer K Quint, Richard Fry, Gwyneth Davies

**Affiliations:** 1 Population Data Science, Swansea University Medical School, Swansea, Wales, UK; 2 School of Public Health, Imperial College London, London, England, UK

**Keywords:** Paediatric asthma, Asthma Mechanisms, Asthma Epidemiology

## Abstract

**Background:**

Asthma morbidity is high among young people, and studies have shown associations between asthma and school attendance and educational attainment. However, findings are unclear concerning associations between air pollution and these educational outcomes, and whether asthma might mediate any associations.

**Objective:**

This review aimed to summarise, and find gaps in, the research on outdoor air pollution, asthma and educational outcomes. To our knowledge, this is the first review to consider the impact of air pollution or asthma, individually or in combination, on the school attendance and educational attainment of children and young people.

**Design:**

This scoping review, using the Preferred Reporting Items for Systematic reviews and Meta-Analyses extension for Scoping Reviews method, reports on searches for English language studies of air pollution, asthma and school attendance and educational attainment in eight databases with tabulation and synthesis of the extracted data.

**Results:**

Association between air pollution and school absence was found to be weaker than for active asthma with this outcome. Uncontrolled asthma was associated with lower educational attainment, but findings on air pollution exposure were mixed. Two studies found associations for air pollution with poorer educational outcomes for young people with asthma. Long-term exposure to air pollution, and an increase in the frequency of peaks of air pollution, were associated with worse educational outcomes. Inequalities in access to healthcare and education were associated with uncontrolled asthma and lower educational outcomes. Only one study used linked health, environmental and educational data.

**Conclusions:**

Linked administrative data will be important to enable longitudinal studies of exceptionally large populations to explore asthma exacerbation, baseline and spikes of air pollution and risk factors. Analyses should control for type of educational assessment and specific particulate exposure. Studies should examine temporal changes and a variety of geographical settings to identify even weak associations to inform approaches to address inequalities of public health and education.

**WHAT IS ALREADY KNOWN ON THIS TOPIC**

- Asthma is a common disease among young people and is associated with worse school attendance and educational attainment, but the impact of air pollution is unclear.

**WHAT THIS STUDY ADDS**

- Studies rarely used anonymised, linked health, environmental and education data. Data gaps were identified in considering the components of particulate air pollution, very large populations and coverage of various geographical areas.

**HOW THIS STUDY MIGHT AFFECT RESEARCH, PRACTICE OR POLICY**

- This review emphasises the need for high dimensionality of data and very large populations in future studies. Such an approach will enable the identification of previously hidden associations.

## Introduction

 The quality of breathed air is of interest because of the impact on physical and mental well-being.^1^,^4^ Air pollution poses a health risk^5^,^9^ since it may worsen respiratory health^10 11^ through allergic responses^12 13^ and damage the respiratory system.^14 15^ The prevalence of asthma among young people is increasing globally, and there are plausible biological mechanisms for effects on the well-being and educational outcomes of young people.^16^

Many studies explore the impact of air pollution or asthma on educational outcomes in the urban environment, but the full variety of environments requires investigation.^2^ While most studies consider the impact of component pollutants in isolation, some also explore the combined effects of specific air pollutants.^1 17^ The impact of air pollution on children and young people is considered to be higher when compared with adults because of their time spent in the outdoor school environment for breaks and exercise,^9 18^ but the impact of the combined exposure to air pollution at school, home and travelling between home and school is not described. Although there is an understanding that asthma can lead to worse attendance at school,^19 20^ there is conflicting evidence concerning the impact of asthma on educational attainment.^21 22^ School age children with severe/frequent asthma exacerbation may miss more school,^23^ which may impact their learning and long-term attainment.^24^,^26^ Recent cross-sectional studies^2 27^ have shown the impact of air pollution in worsening attainment, and that continuing exposure to air pollution damages cognition.^28^ Covariates such as sex, ethnicity and socioeconomic status are important considerations in the design of studies to control for their impact on educational outcomes.^29^ This study used the Preferred Reporting Items for Systematic reviews and Meta-Analyses extension for Scoping Reviews (PRISMA-ScR) to provide a rapid, broad review of this complex question. To our knowledge, this is the first review to consider the impact of air pollution or asthma, individually or in combination, on the school attendance and educational attainment of children and young people.

### Objectives

This scoping review ascertains the current understanding of the associations between asthma and school absence or educational attainment, and between air pollution and school absence or educational attainment. The identification of gaps in research will help to inform the future design of experimental investigations of health, environmental and education data.

### Research question

What is the evidence that, individually or in combination, exposure to air pollution and current asthma are associated with, either or both, the school attendance and educational attainment of children and young people?

## Methods

### Search strategy

This scoping review used the PRISMA-ScR^30 31^ and the Joanna Briggs Institute (JBI) methodology for scoping reviews^32^ ensuring reproducibility and validity. The protocol was registered with the Open Science Framework on 30 July 2024.

A broad range of articles was captured to explore the amount and quality of the literature describing the impact of air pollution or asthma, individually or in combination, on educational outcomes and to describe the methodologies, evidence and gaps in knowledge. Eight academic databases (ASSIA, CINAHL, Cochrane Database of Systematic Reviews, Embase, MEDLINE, Education Research Complete, SCOPUS and Web of Science) were searched. 7959 studies were returned from the database search. After screening, the full review and data extraction was completed on 37 articles included from the database search and 4 articles added from a manual search.

### Identification of relevant studies

A preliminary search of MEDLINE and the Cochrane Database of Systematic Reviews during May 2024 found neither systematic nor scoping reviews addressing air pollution, asthma and school outcomes. Four items were found in a search of MEDLINE for scoping reviews concerning air pollution, asthma and children.^10 14 33 34^ Two of these address children and young people as the population of study,^14 34^ and the other two consider children as part of the population studied.^10 33^

### Inclusion and exclusion criteria

The review considered studies examining any association between air quality or asthma, individually or together and the school attendance or educational attainment of young people, aged 3–19 years. Only peer-reviewed, English language studies published from 2000 to 2024 were considered. The search strategy was developed by CB with advice from the coauthors and consultation with a librarian. An initial search of MEDLINE was conducted to identify further key words, phrases, terminology and subject headings found in the title or abstract of articles. The results of the initial searches were analysed to determine additional search terms used in the full search strategy reproduced in online supplemental I. The age range of 3–19 years was considered to be inclusive of the compulsory education systems of any country. The pollutants searched for were darbon dioxide, nitrogen dioxide, sulfur dioxide, ozone, benzene, nitrate, ammonia, elemental carbon, particulate matter and biological allergens (eg, pollen). These pollutants were included as important exposures following the preliminary literature search and a consideration of air quality data sets. The comparators in the search were made as broad as possible by using any indication of asthma in the population of the study. Finally, the outcomes of school attendance and educational attainment were explored separately before bringing together the results.

### Data extraction and analysis

The search terms applied to the databases yielded 7959 articles on 31 July 2024. The articles returned by the databases were collated in EndNote V.20.6 and then exported to Rayyan to remove duplicate articles, to screen and to review the included studies. The first author (CB) and the peer reviewer (ZG) made all judgments and selections manually and independently. 62 articles were taken forward for a full text review using the judgement tool in online supplemental II. The reason for the exclusion of any article was recorded at the stage of removal from the sample of studies. In the case of disagreement over the inclusion or exclusion of an article, MA reviewed and made a final decision.

### Quality assessment

The quality assessment process was developed from a tool developed by the JBI.^35^ The data extraction tool developed for the review included 20 domains and criteria were assessed as shown in table 1.

**Table 1:** Quality assessment scoring and judgement categories for the reviewed articles

Response to the quality assessment area	Score per area	Total score recorded	Judgement of quality recorded
Yes	1	16–20	High
No	−1	8–15	Satisfactory
Cannot be answered	−1	1–7	Poor
Not applicable	0		

The quality assessment tool provided a structure for the judgement of articles in domains that covered the elements of study design, statistical approach, the quality of definitions, appraisal undertaken and the quality of reporting. The tool is included in online supplemental III.

### Patient and public involvement

Patient and public involvement members were involved in prioritising this research and providing guidance on reporting and dissemination plans. Neither patients nor the public were involved in the design, conduct or reporting of our research.

### Data extraction

**Figure 1:**
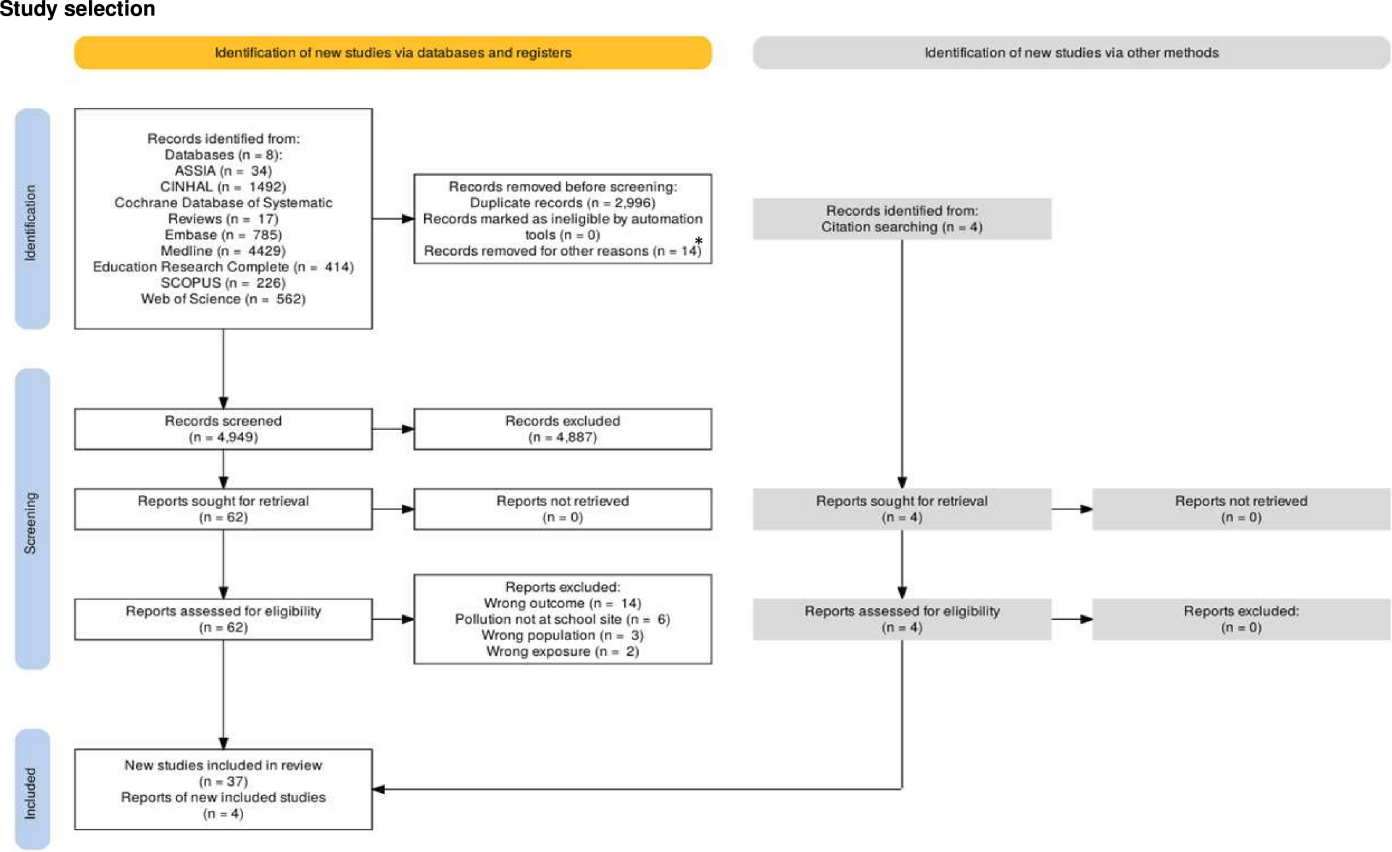
PRISMA diagram of the selection process and reasons for exclusion. *The 14 articles removed for other reasons were article retractions. PRISMA, Preferred Reporting Items for Systematic Reviews and Meta-Analyses.

37 articles from the full text screening, and 4 articles from a manual search in Google Scholar and in the references of the selected articles, were included for data extraction (figure 1). Data extraction used the tool in online supplemental III that was developed using the JBI tool.^35^ The data included the details of publication, the characteristics of the study and the population, the variables considered and the findings that were relevant to the review question. The definitions of air pollution, asthma, attendance and attainment were recorded to aid the comparisons and judgements in the scoping review report. Where available, numerical data representing effect sizes, CIs and probability values were extracted. Quality appraisal was completed for the inclusion of a publication quality appraisal (n=37), publication bias (n=26) and study limitations (n=39).

### Data analysis and presentation

The extracted information was analysed to identify the quality of the articles, key findings on associations, recommendations and knowledge gaps. The results are presented in summary in table 2, and the extracted data is presented in online supplemental IV.

**Table 2:** Overview of the reviewed studies

Summary of included studies (total=41)		
Study design	Experimental	40
	Review	1
Region	Asia	5
	Europe	12
	North America	20
	Oceania	2
	South America	2
Data used	Institutional and surveyed	31
	Routinely collected administrative data	9
Study context	Urban	17
	Urban and semiurban	3
	Urban and rural	16
	Rural	1
	Rural with industry	1
	Unspecified	3
Study period	1 year	20
	2–4 years	10
	More than 5 years	10
Variables	Asthma	31
	Air pollution	17
Outcomes	Educational absence	22
	Educational attainment	12

## Results

41 papers were included in this review, 37 from the planned search and 4 from a manual search. The articles were published between 2001 and 2024 and were conducted in Australia (n=1), Brazil (n=2), Canada (n=1), China (n=1), Indonesia (n=1), Italy (n=1), Japan (n=1), South Korea (n=1), New Zealand (n=1), Qatar (n=1), Sweden (n=4), UK (n=7), USA (n=18) and the scoping review led in Canada (n=1).

### Quality assessment

The quality of the studies was judged to be satisfactory (n=19) and high (n=22) because of the good study design, use of appropriate analytical tools and effective structure and content of reporting. A summary of the quality assessment is shown in table 3.

**Table 3:** A summary of the quality assessment

Judgement	Score	Number of articles in the category	Range of score in the category
High	16–20	22	16–19
Satisfactory	8–15	19	8–15
Poor	1–7	0	Not applicable

### Contexts and designs

Most studies were conducted in urban (n=17), or urban and semiurban (n=3) environments. There were studies across urban and rural (n=16) environments, often involving nationwide surveys of health or satellite observation of air pollution. Rural geography (n=1) and rural with industry (n=1) were much less common locations for study. There were also studies with unspecified geographies of study (n=3) which included the systematic review. The use of linked health data was uncommon (n=9) with the use of routinely collected health, environment and education data (n=6) being rare. Almost half of the articles (n=20) reported studies for a single year of data, with the number of studies using a longitudinal period of more than 5 years being low (n=10). Except for the systematic review, all the studies employed mathematical analysis of the collected data and most reported effect sizes with CIs. The studies were balanced between cohort (n=18) and cross-sectional (n=22) studies and equally distributed between contemporary (n=20) and retrospective (n=20) studies.

### Exposure and outcome variables

Most articles considered asthma as the only or the main exposure (n=31) with these being divided into those which recorded asthma status through surveys of parents, young people or schools (n=22) and those that used routine medical records (n=9). Air pollution was considered as an exposure singly or in combination with asthma status (n=17), with most of these employing ground measurements of air pollution and satellite measurement for the remaining study (n=1). The studies reporting on absence from school (n=24) drew on data from parents, schools or educational districts. Most of the studies examining attainment (n=22) reported at school level with some exploring attainment (n=12) through individual testing of young people and some analysing individual attainments in national assessments.

### Summary of results

Most of the studies considered in this review were conducted wholly or partly in urban environments and considered asthma as the exposure. The use of routinely collected and linked individual data for health, environment or education was restricted to a few studies.^216 21 22 36^,^40^ Only one study used linked health, environmental and educational data.^2^ Many of the studies were cross-sectional and considered a single year of data which did not explore long-term impacts. Four articles considered air pollution and asthma together, with asthma defining the population,^2^ as a covariate,^41^ and outcomes of respiratory disorders.^9 42^ Association between air pollution and school absence was found to be weaker than for active asthma. Uncontrolled asthma was associated with lower educational attainment, but findings on air pollution exposure were mixed. Two studies found associations for air pollution with poorer educational attainment^2^ or school attendance^41^ for young people with asthma. Long-term exposure to air pollution and an increase in the frequency of peaks of air pollution were associated with worse educational outcomes.

## Discussion

Studies have shown that uncontrolled asthma is associated with worse educational outcomes. However, the mixed findings for associations between air pollution and educational outcomes do not consider the effect of specific particulates, the impact of physical geography, changes over time and small effect sizes. Very few studies considered rural air quality for which sources, concentrations and distributions of pollutants differ from urban environments, but the impacts on health and education particular to rural environments are not unimportant. Work has been undertaken to explore the impact of asthma and air pollution on under-resourced groups. There has been very little study of the impact on the emotional and cognitive abilities of young people. Examination of linked health, environmental and educational data was limited to one study.

Articles consistently reported an association between asthma and absence. The identification of school absences by parents differed from school records, with both under and over parental estimation being found.^43^ The association of asthma and school absence greater than the school target for absence is strongest for younger students^44^ for whom lower body mass, higher respiratory rate and susceptibility to respiratory illness were suggested as reasons for the increased morbidity. Analyses of asthma show a stronger association with school absence for young people with poorly controlled or uncontrolled asthma,^20 45 46^ and those with the most severe asthma are likely to have the greatest excess of school absence.^47^ Asthma was found to account for more school absence than any other chronic disease,^21^ but evidence suggests that asthma does not impact lateness of arrival at school.^48^ The reduced well-being of young people due to asthma leads to more school absence and lower engagement in physical education, but excess school absence is minimised by effective health and educational support of young people.^45^ Cognitive and emotional comorbidities are important covariates with asthma.^49^ Longitudinal studies of national populations with international comparisons by multidisciplinary teams will be necessary to account for small effects and covariates.^50^

Asthma and lower educational attainment were associated. Suggested mechanisms included lower school attendance,^51 52^ drowsiness due to medication,^53^ worse cognition,^22^ reduced physical well-being and less engagement with school.^54^ Deficits in attainment were found to increase for less controlled and more severe asthma.^51^ Worse academic performance was found to be compounded across multiple years, with 58.0% (95% CI 21% to 78%) lower odds of proficiency on Mathematics Measure of Academic Progress.^55^ The association between asthma and lower attainment is strongest for ethnic minority groups.^27^ However, in other communities, the size of the association is small and below statistical significance,^56^ or weak even for active asthma.^38^ Elsewhere, it has been shown that only hospitalisation for asthma is associated with lower attainment,^21 23^ the strongest association being for fine particulate matter with a diameter of 2.5 micrometers or less (PM_2.5_) exposure with hospitalisation for respiratory conditions.^39^ Indeed, a study of a population with good health access showed educational attainment to be similar for young people with and without asthma.^37^ One article reported slightly better performance for young people with controlled asthma, even for controlled severe asthma, and suggested that family factors are important.^36^ It is also important to control for the effects of additional learning and other health needs which may impact attainment.^16 57^ Effective support mitigates inequalities in attainment, for example, the deficits in attainment of children entering education with asthma.^58^ Thus, the complex circumstances and needs of young people need further examination.

Chronic and acute exposure to air pollution was both found to be associated with school absence.^59 60^ One study discussed indoor air pollution as a covariable in explaining the lower school attendance for females with asthma, with a risk ratio of 1.66 (95% CI 1.19 to 2.31) being reported for increased risk of missed school days.^61^ PM_2.5_ was identified as the air pollutant most strongly associated with absenteeism, but O_3_ appears to impact young people who are susceptible to infectious disease.^40 61^ PM_2.5_ was associated with absence more than O_3_.^62^ However, in one study, while exposure to CO, and more strongly O_3_, was associated with greater school absence,^63^ PM_10_ was negatively associated with absence. A high altitude, desert environment and complex makeup of PM_10_ were suggested as important variables of environmental exposure.^63^ The geography of locations and socioeconomic patterns was suggested as important differences in the exposure to types and concentrations of air pollutants.^62^ Complexity is also found in the association between PM_2.5_ and school absence, with higher absence found only for schools serving communities with a high percentage of young people in the lowest socioeconomic status groups.^52^ Schools serving more socioeconomically disadvantaged communities were found to be in more polluted locations.^42^ So, the reduction of ambient air pollutant concentrations would benefit low socioeconomic status groups most.^42 62^ Further studies should examine chronic and acute exposure and specific types of PM_2.5_.

The findings concerning associations between air pollution exposure and educational attainment were not consistent and this may be explained by the differences in study types and locations. However, exposure to NO_2_ was associated with lower individual educational attainment even when adjustment was made for individual and household risk factors, and for pollen and other air pollutants with a unit (10 µg/m3) increase of short-term exposure to NO_2_ was associated with 0.044 (95% CI 0.079 to 0.008) reduction of standardised Capped Point Score.^2^ The school level attainment also worsens as NO_2_ and PM_2.5_ concentrations increase.^64^ The highest levels of concentration of NO_2_ are associated with the greatest decreases in attainment.^42^ Even with control for socioeconomic deprivation, the frequency of peak air pollution is associated with lower attainment in mathematics, with a similar trend for English attainment, though it was not found to be statistically significant.^65^

A further longitudinal study, including following individuals from birth to adulthood, is necessary. Differences in the definition of asthma, the measurement of attainment and mixed findings^21 36 66^ made meta-analysis unfeasible. Those articles that explore attainment do so variously for the assessment of skills, knowledge and understanding, making comparison difficult. It may be that school absence affects the assessment of skills less than the assessment of knowledge because there are more opportunities to develop the skills during periods of attendance.^21 66 67^ The attitudes of the community and young people to education, and the worse quality of well-being,^66 68^ are covariates which may be associated with lower educational outcomes. The capacity of young people to respond to increases in the concentration of air pollution,^65^ and to complex social stressors,^9^ was identified as an issue of inequality in which groups of young people in the lowest levels of socioeconomic status are impacted more by chronic exposure and those groups in the highest levels of socioeconomic status are more strongly impacted by acute exposure. Studies of large populations in diverse environments are needed to explore issues of ethnicity, low resourcing of communities and the geography of locations.^69^

### Strengths and limitations

The use of a detailed search protocol and its application to eight databases yielded a useful number of medium-to-high quality articles. These databases cover journals that specialise in health, the environment and education. Following the PRISMA-ScR process and tracking the review with peer reviewers ensured a robust process. Only English language articles were reviewed, and the date range of the search was 2000–2024, which limited the studies that were considered. Additionally, the article search was not run again after July 2024 and so articles published from August 2024 to July 2025 have not been reviewed. This scoping review did not explore the impact of indoor pollution which has a baseline of the ambient outdoor pollution. However, the result of time spent inside a house, where cooking and heating may raise the exposure to particulate matter, NO_2_ and CO. This exposure to indoor air pollution has detrimental effects on lung function, asthma and educational outcomes.^61 68^ There were some limits to the comparisons of the papers because of the variety of exposures, outcomes, analytical processes and the data sources, and a meta-analysis was not possible. From 2000 to 2024, the use of anonymised, routinely collected, linked health data has increased and these studies are distinct from the single year, small population studies.

## Conclusion

Currently, there is incomplete knowledge concerning the mechanisms by which air pollution may impact the educational outcomes of young people. Longitudinal population studies will help to inform policy and approaches to support provided by health, government and educational institutions, and commerce. The different impacts of chronic and acute air pollution exposure have been studied, but the long-term effects in different geographies and social conditions are not fully described. Further exploration of the impact of air pollution and asthma on the outcomes from different educational learning experiences and assessment styles is necessary.

To meet these gaps in understanding, studies should use longitudinal and very large populations with international comparison. Studies should be conducted across a range of geographies and consider the specific particulate air pollutants. The use of anonymised, linked health, environmental and educational data will provide reliable datasets and enable analyses of high statistical power. Examination of the development of cognition and attainment for individuals across time will improve our understanding of the long-term impacts of air pollution and asthma.

## Supplementary material

10.1136/bmjresp-2025-003527 online supplemental file 1

10.1136/bmjresp-2025-003527 online supplemental file 2

10.1136/bmjresp-2025-003527 online supplemental file 3

10.1136/bmjresp-2025-003527 online supplemental file 4

## Data Availability

All data relevant to the study are included in the article or uploaded as supplementary information.
