# Supplementary material for: Impact of air pollution and asthma on school attendance and educational attainment: a scoping review
Source: BMJ Open Respir Res. 2025 Dec 7;12(1):e003527. doi: 10.1136/bmjresp-2025-003527 (PMC12684173; doi:10.1136/bmjresp-2025-003527)
Supplement: online supplemental file 1 [file bmjresp-12-1-s001.docx]

**Supplemental I:**

Searches

## Searches in Medline, Education Research Complete and CINAHL.

*Database coverage: 2000 to 2024* *Date Last Searched: July 31st, 2024*

1. Terms

Population: Children.

**TI (Child* OR Adolescent* OR Youth* OR Teenage* OR Education* OR "School Age*" OR School* OR Student* OR "3-19") OR AB (Child* OR Adolescent* OR Youth* OR Teenage* OR Education* OR "School Age*" OR School* OR Student* OR "3-19")**

Exposure: Air Quality.

**TI ( Air Quality OR Air contamination OR Air Pollution OR Pollut* OR “Pollution Event” OR Allergen* OR “Particulate Matter*” OR Nitro* OR Sulfur* OR Sulphur* OR Ammon* OR Benz* OR Temperature ) OR AB ( Air Quality OR Air contamination ORAir Pollution OR Pollut* OR “Pollution Event” OR Allergen* OR “Particulate Matter*” OR Nitro* OR Sulfur* OR Sulphur* OR Ammon* OR Benz* OR Temperature)**

Comparison: Asthma.

**TI (Asthma* OR Wheez* OR "Acute Asthma*" OR "Asthma* Attack*" OR "Asthma* Exacerbation*” OR "Asthma* Complication*" OR Respirat*) OR AB (Asthma* OR Wheez* OR "Acute Asthma*" OR "Asthma* Attack*" OR "Asthma* Exacerbation*” OR "Asthma* Complication*" OR Respirat*)**

Outcome1: School attendance.

**TI ( “School Attend*” OR “School Absen*” OR “School Non-Attend*” ) OR AB ( “School Attend*” OR “School Absen*” OR “School Non-Attend*” )**

Outcome2: School attainment.

**TI ( “Educational Assess*” OR “Educational Attain*” OR “Educational Achieve*” OR "Cogniti* Abilit*" OR Cogniti* ) OR AB ( “Educational Assess*” OR “Educational Attain*” OR “Educational Achieve*” OR "Cogniti* Abilit*" OR Cogniti*)**

The complete search combined the sub-searches as:

**(Population AND (Exposure OR Comparison) AND (Outcome 1 OR Outcome 2))**

1. MeSH Subject headings.

Population: Children.

**(MH "Child+") OR (MH "Adolescent")**

Exposure: Air Quality.

**(MH "Air Pollution+") OR (MH "Air+/AN") OR (MH "Air Pollutants+")**

Comparison: Asthma.

**(MH "Asthma, Exercise-Induced") OR (MH "Cough-Variant Asthma") OR (MH "Asthma+"))**

Outcome: Education.

**(MH "Schools+")**

The complete search with MeSH subject headings combined the sub-searches as:

**(Population AND (Exposure OR Comparison) AND Outcome))**
