# Supplementary material for: Impact of air pollution and asthma on school attendance and educational attainment: a scoping review
Source: BMJ Open Respir Res. 2025 Dec 7;12(1):e003527. doi: 10.1136/bmjresp-2025-003527 (PMC12684173; doi:10.1136/bmjresp-2025-003527)
Supplement: online supplemental file 2 [file bmjresp-12-1-s002.docx]

## **Supplemental II:**

## Peer Review Tool For The Selection of Articles

|  | Guidance | Description or information |
| --- | --- | --- |
| Reference |  |  |
| Publication date | 01/01/2020 onwards |  |
| Country of study | Any |  |
| Language | English |  |
| Peer Reviewed article | Yes |  |
| Attendance/absence defined | Yes |  |
| Attainment/achievement/  Assessment defined | Yes |  |
| Study design | Any type |  |
| Analysis | Any type |  |
| Population | Ages 3 – 19 years |  |
| Variable explored | Air pollution |  |
| Variable explored | Asthma |  |
| Outcomes | School or education |  |
| Strengths | Discussed |  |
| Limitations | Discussed |  |
| Conclusions | Discussed |  |
| Recommendations | Discussed |  |
| Quality appraisal by the authors | Discussed |  |
| Conflict of Interest | Stated |  |
|  |  |  |
| Inclusion in the review | Yes/No |  |
