# Supplementary material for: Impact of air pollution and asthma on school attendance and educational attainment: a scoping review
Source: BMJ Open Respir Res. 2025 Dec 7;12(1):e003527. doi: 10.1136/bmjresp-2025-003527 (PMC12684173; doi:10.1136/bmjresp-2025-003527)
Supplement: online supplemental file 3 [file bmjresp-12-1-s003.docx]

## **Supplemental III:**

## Data Extraction Tool

|  | Guidance | Description or information | Score |
| --- | --- | --- | --- |
| Reference |  |  |  |
| Year |  |  |  |
| A priori Design | Present |  |  |
| Comprehensive search | Completed |  |  |
| Study selection and data extraction | Described |  |  |
| Gray Literature | Included/excluded |  |  |
| Study Characteristics | Described |  |  |
| Effect sizes | Included |  |  |
| Confidence intervals | Included |  |  |
| Probability scores | Included |  |  |
| Strengths | Described |  |  |
| Limitations | Described |  |  |
| Population definitions | Included |  |  |
| Exposure definition | Included |  |  |
| Comparator definitions | Included |  |  |
| Outcomes definitions | Included |  |  |
| Quality appraisal by the authors | Included |  |  |
| Conclusion | Included |  |  |
| Recommendations | Included |  |  |
| Pooling Methods | Included |  |  |
| Publication Bias | Included |  |  |
| Conflict of Interest | Included |  |  |
| Quality Score | Maximum = 20 |  |  |
| Overall Quality | High/Satisfactory/  Poor |  |  |
|  |  |  |  |
| Response | Score | Judgement | Score |
| Yes | 1 | High | 16 to 20 |
| No | -1 | Satisfactory | 8 to 15 |
| Cannot be answered | -1 | Poor | 1 to 7 |
| Not applicable | 0 |  |  |
