# Supplementary material for: Impact of air pollution and asthma on school attendance and educational attainment: a scoping review
Source: BMJ Open Respir Res. 2025 Dec 7;12(1):e003527. doi: 10.1136/bmjresp-2025-003527 (PMC12684173; doi:10.1136/bmjresp-2025-003527)
Supplement: online supplemental file 4 [file bmjresp-12-1-s004.docx]

**Supplemental IV:** Characteristics of included studies

| Authors | Title | | | Year | | Region | | Country | | Environment | | Contemporary/Retrospective | | Cohort/ Cross-section | | Duration | | | Findings | | Association Found | Use of linked health data | Quality Score | Overall Quality |
| --- | --- | --- | --- | --- | --- | --- | --- | --- | --- | --- | --- | --- | --- | --- | --- | --- | --- | --- | --- | --- | --- | --- | --- | --- |
| BENER, A., KAMAL, M. & SHANKS, N. J. | Impact of asthma and air pollution on school attendance of primary school children: are they at increased risk of school absenteeism? The Journal of asthma: official journal of the Association for the Care of Asthma, 44, 249-252. | | | 2007 | |  | | Qatar | | Urban and semi-urban | | Contemporary | | Cross-section | | 1 | | | Absence due to asthma p = 0.036. 8 days abs asthma, 4days non asthma, 7 days wheeze. | | Asthma -> Absence | None | 8 | Satisfactory |
| BONILLA, S., KEHL, S., KWONG, K. Y. C., MORPHEW, T.,  KACHRU, R. &  JONES, C. A. | School absenteeism in children with asthma in a Los Angeles inner city school. The Journal of pediatrics, 147, 802-806. | | | 2005 | | Los Angeles | | USA | | Urban and semi-urban | | Contemporary | | Cross-section | | 1 | | | Overall, students with known asthma missed 2 more total days/year and 1.4 more days/year because of respiratory symptoms compared with children with either low or high probability of asthma (P < .01). | | Asthma -> Absence | None | 12 | Satisfactory |
| CARTER-POKRAS, O. D., BUGBEE, B. A., GOLD, R. S.,  LAUVER, P. E., AIKEN, R. & ARRIA, A. M. | Utilizing Student Health and Academic Data: A County-Level Demonstration Project. Health promotion practice, 22, 193-203. | | | 2021 | | Maryland | | USA | | Rural | | Contemporary | | Cross-section | | 1 | | | Having asthma or a mental health diagnosis was positively associated with absences. | | Asthma -> Absence | None | 13 | Satisfactory |
| CHEN, L., JENNISON, B. L., YANG, W. & OMAYE, S. T. | Elementary school absenteeism and air pollution. Inhalation toxicology, 12, 997-1016. | | | 2000 | | Washoe County | | USA | | Urban and Rural | | Retrospective | | Cross-section | | 2 | | | For every 1.0 ppm and 50 ppb increase in CO and O3 , the absence rate would increase 3.79% (95% CI 1.04–6.55%) and 13.01% (95% CI 3.41–22.61%), respectively. However, PM10 values were negatively correlated with school absenteeism. | | Air Pollution -> Absence | None | 8 | Satisfactory |
| CONTE KEIVABU, R. & RÜTTENAUER, T. | London congestion charge: the impact on air pollution and school attendance by socioeconomic status. Population and Environment, 43, 576-596. | | | 2022 | | London | | UK | | Urban | | Retrospective | | Cohort | | 7 | | | These overall estimates thus lead us to conclude that the CCZ policy did not substantially affect school absences. PM2.5 increases absences for low-SES schools but not for the other SES groups or the pooled sample. The elasticity observed suggests an increase of absences by 1.27 percentage points per 1 µg/m3 increase in PM2.5 air pollution. | | Air Pollution -> Absence | None | 16 | High |
| EVANS, A.,  FAREWELL, D.,  DEMMLER, J., BANDYOPADHYAY, A., POWELL, C. V. E. & PARANJOTHY, S. | Association of asthma severity and educational attainment at age 6-7 years in a birth cohort: population-based record-linkage study. Thorax, 76, 116-125. | | | 2021 | | Wales | | UK | | Urban and rural | | Retrospective | | Cross-section | | 7 | | | Following adjustment for social deprivation, birth and school characteristics, only asthma inpatient hospital admission remained associated with increased risk for not attaining the expected level at KS1 (aOR 1.14 95% CI (1.02 to 1.27)). | | Asthma admission -> Lower Attainment | Yes | 18 | High |
| FLEMING, M., FITTON, C. A.,  STEINER, M. F. C., MCLAY, J. S., CLARK, D., KING, A., MACKAY, D. F. & PELL, J. P. | Educational and health outcomes of children treated for asthma: Scotland-wide record linkage study of 683 716 children. The European respiratory journal, 54, | | | 2019 | | Scotland | | UK | | Urban and rural | | Retrospective | | Cohort | | 4 | | | The 45,900 (6.0%) children treated for asthma had an increased risk of hospitalisation, particularly within the first year of treatment (IRR 1.98, 95% CI 1.93-2.04), and increased mortality (HR 1.77, 95% CI 1.30-2.40). They were more likely to have special educational need for mental (OR 1.76, 95% CI 1.49-2.08) and physical (OR 2.76, 95% CI 2.57-2.95) health reasons, and performed worse in school exams (OR 1.11, 95% CI 1.06 1.16). Higher absenteeism (IRR 1.25, 95% CI 1.24-1.26) partially explained their poorer attainment. | | Treated asthma -> Lower Educational Outcomes | Yes | 14 | Satisfactory |
| JUBER, N. F.,  SHONGWE, M. C.,  MA, E., LIN, W.-S. & NGO, T.-H. | The Association Between Pediatric Asthma and Missed School Days: A Sex-Stratified Secondary Analysis From the Indonesian Family Life Survey. Asia-Pacific Journal of Public Health, 85-87. | | | 2023 | | Indonesia | | Indonesia | | Urban and rural | | Contemporary | | Cross-section | | 1 | | | Pediatric asthmatic females had an increased risk of missed school days in the adjusted model (RR = 1.66, 95% CI [1.19, 2.31]). In addition, female asthmatics diagnosed at age 0 to 5 years had a significantly increased risk of missed school days (RR = 1.57, 95% CI [1.05, 2.36]). | | Asthma -> absence | None | 10 | Satisfactory |
| Authors | Title | | | Year | | Region | | Country | | Environment | | Contemporary/Retrospective | | Cohort/ Cross-section | | Duration | | | Findings | | Association Found | Use of linked health data | Quality Score | Overall Quality |
| KIM, C. H., GEE, K. A. & BYRD, R. S. | Excessive Absenteeism Due to Asthma in California Elementary Schoolchildren. Academic Pediatrics, 20, 950-957. | | | 2020 | | California | | USA | | Urban and rural | | Retrospective | | Cross-section | | 3 | | | 715 respondents represent an estimated 314,200 California school children with asthma. 50.3% of students missed ≥1day, and 11.7% missed ≥9 days of school due to asthma. Odds of EA were significantly higher for younger children, lower-income families, and rural students, but not significant for any absenteeism. Indicators of greater asthma severity and poorer control were significantly associated with both EA and any absenteeism. | | Asthma -> absence | None | 18 | High |
| KIM, S. H., SOHN, K. H.,  KANG, S. Y., KIM, J. H., KWON, J. W., NA, J. I., KIM, B. K.,  CHANG, Y. S. &  CHO, S. H. | School Absenteeism Associated with Asthma and Allergic Diseases in Korean School-Aged Children. Pediatric Allergy Immunology and Pulmonology, 31, 151-157. | | | 2018 | | Gyeonggi | | South Korea | | Urban and Rural | | Contemporary | | Cross-section | | 1 | | | Subjects with lifetime diagnosis of asthma had a significantly higher odds of missing school when they had recently experienced rhino conjunctivitis symptoms [adjusted odds ratio (OR)=1.58; 95% confidence interval (CI)=1.07–2.34; P=0.022] or ever received treatment for rhinitis in the past 12 months (adjusted OR=1.41; 95% CI=1.04–1.92; P=0.028). | | Asthma Exacerbation -> Absence | None | 10 | Satisfactory |
| KOHEN, D. E. | Asthma and school functioning. Health reports, 21, 35-45. | | | 2010 | | Canada | | Canada | | Urban and Rural | | Retrospective | | Cross-section | | 1 | | | Compared with children who did not have chronic conditions, those with asthma tended to perform less well, and those with the most severe asthma had the poorest outcomes. | | Asthma -> Lower attainment | None | 14 | Satisfactory |
| KOINIS-MITCHELL, D., KOPEL, S. J., FARROW, M. L.,  MCQUAID, E. L. & NASSAU, J. H. | Asthma and academic performance in urban children. Annals of allergy asthma & immunology: official publication of the American College of Allergy Asthma & Immunology, 122, 471-477. | | | 2019 | | Northeast | | USA | | Urban | | Contemporary | | Cross-section | | 1 | | | Finally, significant associations were found between asthma and academic indicators, stratified by ethnicity. Among Latinos, better lung function was associated with less careless schoolwork (t¼ 2.38, P¼.02), as well as higher WRAT math (t¼ 2.73, P¼.01) and reading scores (t¼2.98, P¼.003). Furthermore, higher FEV variability was associated with worse WRAT math (t¼ 2.20, P¼.02), and reading scores (t¼ 2.19, P¼.03). | | Asthma -> Lower attainment | None | 12 | Satisfactory |
| LIBERTY, K. A., PATTEMORE, P., REID, J.,  TARREN-SWEENEY, M., | Beginning school with asthma independently predicts low achievement in a prospective cohort of children. CHEST, 138, 1349-1355. | | | 2010 | | Christchurch | New Zealand | | | Urban and semi-urban | | Contemporary | | Cross-section | | 1 | | | Children who entered school with asthma were more likely to be 6 months behind other participants in reading words (P 5 .023) and books (P 5 .026), but not in math (P 5 .167) at the end of the first year of school. Achievement was not related to asthma severity. | | Asthma -> Lower attainment | None | 18 | High |
| LUNDHOLM, C.,  BREW, B. K., D'ONOFRIO, B. M., OSVALD, E. C.,  LARSSON, H. &  ALMQVIST, C. | Asthma and subsequent school performance at age 15-16 years: A Swedish population-based sibling control study. Scientific reports, 10, 7661 | | | 2020 | | Sweden | Sweden | | | Urban and rural | | Retrospective | | Cohort | | 5 | | Children with asthma and severe asthma performed slightly better in school than children without asthma when adjusting for measured confounders, but the associations were attenuated in sibling comparisons. In contrast, children with uncontrolled asthma performed slightly worse (e.g. Grade 9: βadj = −9.9; 95% ci −12.8 to −7.0; Cohen’s d = 0.16). This association remained for uncontrolled asthma in Grade 9 in sibling comparisons (Grade 9: β = −7.7 points; 95% CI −12.6 to −2.6; Cohen’s d = 0.12), but not for Grades 7–8. The attenuation of estimates when controlling for familial factors using sibling comparisons suggests that the differences were due to familial factors, rather than being causal. The remaining associations in sibling comparisons between uncontrolled asthma in Grade 9 and school performance are consistent with a causal association. | | | Asthma Uncontrolled -> Lower Attainment | Yes | 18 | High |
| Authors | Title | | | Year | | Region | Country | | | Environment | | Contemporary/Retrospective | | Cohort/ Cross-section | | Duration | | Findings | | | Association Found | Use of linked health data | Quality Score | Overall Quality |
| MAKINO, K. | Association of school absence with air pollution in areas around arterial roads. Journal of epidemiology, 10, 292-299. | | | 2000 | | Tokyo | Japan | | | Urban | | Retrospective | | Cohort | | 4 | | The prevalence of absence was significantly correlated with suspended particulate matter (SPM), nitrogen dioxide and atmospheric temperature in school A. SPM, nitrogen dioxide, and relative humidity were correlated positively, and atmospheric temperature was negatively in some significant cases. | | | Air pollution -> absence | None | 10 | Satisfactory |
| MARCON, A.,  PESCE, G.,  GIRARDI, P.,  MARCHETTI, P.,  BLENGIO, G.,  SAPPADINA, S. D., FALCONE, S.,  FRAPPORTI, G., PREDICATORI, F. & DE MARCO, R. | Association. between PM10 concentrations and school absences in proximity of a cement plant in northern Italy. International Journal of Hygiene and Environmental Health, 217, 386-391. | | | 2014 | | Italy | Italy | | | Rural with industry | | Retrospective | | Cohort | | 3 | | An average 10 g/m3increase of PM10 concentration in the previous days (lag0–4) was associated with a statistically significant 2.5% (95%CI: 1.1–4.0%) increase in the rate of school absences. The highest increase in the absence rates (2.4%; 95%CI: 1.2–3.5%) was found 2 days after exposure (lag2). These findings provide epidemiological evidence of the acute health effects of PM10in areas with annual concentrations that are lower than the legal European Union limit of 40 g/m3, and support the need to establish more restrictive legislative standards. | | | Air Pollution -> Absence |  | 16 | High |
| MENDOZA, D. L.,  PIROZZI, C. S.,  CROSMAN, E. T.,  LIOU, T. G., ZHANG, Y., CLEEVES, J. J., BANNISTER, S. C., ANDEREGG, W. R. L. & ROBERT, P III. | Impact of low-level fine particulate matter and ozone exposure on absences in K-12 students and economic consequences. Environmental research letters: ERL [Web site], 15, | | | 2020 | | Salt Lake City | USA | | | Urban | | retrospective | | Cohort | | 3 | | Pollution exposure was associated with a rate ratio as high as 1.02 absences per µg m−3 and 1.01 per ppb increase for PM2.5 and ozone, respectively. Significantly, even PM2.5 and ozone exposure below the air quality index breakpoints for good air quality (<12.1 µg m−3 and <55 ppb, respectively) was associated with positive rate ratios of absences: 1.04 per µg m−3 and 1.01 per ppb increase, respectively. | | | Air Pollution -> Absence |  | 12 | Satisfactory |
| MILLARD, M. W., JOHNSON, P. T.,  HILTON, A., HART, M., | Children with asthma miss more school: fact or fiction? CHEST, 135, 303-306. | | | 2009 | | Dallas | USA | | | Urban | | Retrospective | | Cross-section | | 1 | | Students with asthma in the DISD miss no more school than their classmates without asthma. For school-aged children, untreated asthma can lead to excessive school absences and an inability to maximize their learning experiences when they are at school. | | | Non-Controlled Asthma -> Absence | None | 8 | Satisfactory |
| MITCHELL, R. J., MCMAUGH, A.,  HOMAIRA, N., LYSTAD, R. P., BADGERY-PARKER, T. & CAMERON, C. M. | The impact of childhood asthma on academic performance: A matched population-based cohort study. Clinical and experimental allergy: journal of the British Society for Allergy and Clinical Immunology, 52, 286-296. | | | 2022 | | New South Wales | Australia | | | Urban and rural | | Retrospective | | Cohort | | 13 | | Educational attainment is worse for young people hospitalized with asthma compared to matched peers. | | | Asthma Hospitalisation -> Lower Attainment | Yes | 19 | High |
| MIZAN, S. S.,  SHENDELL, D. G. & RHOADS, G. G. | Absence extended absence and repeat tardiness related to asthma status among elementary school children. The Journal of asthma: official journal of the Association for the Care of Asthma, 48, 228-234. | | | 2011 | | Atlanta | USA | | | Urban | | Contemporary | | Cross-section | | 1 | | Using ANOVA we found that the mean days of absence among students with doctor-diagnosed asthma was 2.72 days compared with 1.89 days for those students without asthma (p=.004). Analys is by race yielded a mean value of 2.03 days for blacks compared with 1.67 days for whites, 1.69 days for Asians, and 2.73 days for mixed race children (p=.20). | | | Asthma -> Absence | None | 14 | Satisfactory |
| MIZEN, A.,  LYONS, J.,  MILOJEVIC, A.,  DOHERTY, R.,  WILKINSON, P., CARRUTHERS, D., AKBARI, A.,  LAKE, I.,  DAVIES, G. A.,  AL SALLAKH, M.,  FRY, R.,  DEARDEN, L. &  RODGERS, S. E. | Impact of air pollution on educational attainment for respiratory health treated students: A cross sectional data linkage study. Health and Place, 63. | | | 2020 | | Cardiff, Wales | UK | | | Urban | | Retrospective | | Cross-section | | 6 | | A unit (10μg/m3) increase of short-term exposure to NO2 was associated with 0.044 (95% CI: 0.079, 0.008) reduction of standardised Capped Point Score (CPS) after adjusting for individual and household risk factors for 18,241 students. This association remained statistically significant after controlling for other pollutants and pollen. | | | Air Pollution -> Lower Attainment | Yes |  | High |
| Authors | Title | | | Year | | Region | Country | | | Environment | | Contemporary/Retrospective | | Cohort/ Cross-section | | Duration | | Findings | | | Association Found | Use of linked health data | Quality Score | Overall Quality |
| MOHAI, P., KWEON, B. S., LEE, S. & ARD, K. | Air pollution around schools is linked to poorer student health and academic performance. Health Affairs, 30, 852-862. | | | 2011 | | Michigan | USA | | | Urban and rural | | Contemporary | | Cohort | | 1 | | Schools located in areas with the highest air pollution levels had the lowest attendance rates—a potential indicator of poor health—and the highest proportions of students who failed to meet state educational testing standards. | | | Air pollution -> Absence | None | 8 | Satisfactory |
| MOONIE, S.,  CROSS, C. L., GUILLERMO, C. J. & GUPTA, T. | Grade retention risk among children with asthma and other chronic health conditions in a large urban school district. Postgraduate medicine, 122, 110-115. | | | 2010 | | Las Vegas | USA | | | Urban | | Contemporary | | Cohort | | 1 | | Lastly, those with asthma (aOR, 1.5) or asthma plus another health condition (aOR, 1.6) were at significantly increased odds of missing more than 10 school days per year compared with healthy students or those with a health condition other than asthma. | | | Asthma -> Absence | None | 17 | High |
| MOONIE, S.,  STERLING, D. A.,  FIGGS, L. W. & CASTRO, M. | The relationship between school absence academic performance and asthma status. Journal of School Health, 78, 140-148. | | | 2008 | | Missouri | USA | | | Urban | | Contemporary | | Cross-section | | 1 | | The highest mean absenteeism was among those with persistent asthma scoring below Nearing Proficient (x = 13.3 ± 10.2 days) | | | Absence -> Lower Attainment | None | 18 | High |
| MOONIE, S. A.,  STERLING, D. A.,  FIGGS, L. & CASTRO, M. | Asthma status and severity affects missed school days. Journal of School Health, 76, 18-24. | | | 2006 | | Louisianna | USA | | | Urban | | Contemporary | | Cross-section | | 1 | | Children with asthma are absent from school more often compared to their healthy peers and this appears to be driven by the underlying severity of symptoms. | | | Asthma severity -> Absence | None | 18 | High |
| MULLEN, C., GRINESKI, S. E.,  COLLINS, T. W. & MENDOZA, D. L. | Effects of PM2.5 on third grade students’ proficiency in math and English language arts. International Journal of Environmental Research and Public Health, 17, 1-23. | | | 2020 | | Salt Lake City | USA | | | Urban and rural | | Contemporary | | Cohort | | 1 | | While higher levels of chronic air pollution were associated with higher percentages of children who tested below proficient in math (p < 0.001), the effect became statistically insignificant with the addition of school disadvantage (Model 3). (page 10) | | | Socioeconomic status -> Attainment | None | 18 | High |
| NILSSON, S.,  ÖDLING, M.,  ANDERSSON, N., BERGSTRÖM, A. &  KULL, I. | Does asthma affect school performance in adolescents? Results from the Swedish population-based birth cohort BAMSE. Pediatric allergy and immunology: official publication of the European Society of Pediatric Allergy and Immunology, 29, 174-179. | | | 2018 | | Country Wide | Sweden | | | Urban and rural | | Contemporary | | Cohort | | 1 | | A statistically significant association for performing less well was seen for ever asthma (ORadj = 1.43, 95% CI = 1.09- 1.88). In analyses of asthma onset, an association was seen for school- age onset (ORadj = 1.49, CI = 1.02- 2.16) and a tendency for persistent asthma (ORadj = 1.61, CI = 0.98- 2.66), although with overlapping confidence intervals. Further, adolescents with uncontrolled asthma tended to perform less well (ORadj = 2.60, CI = 0.87- 7.80) compared to adolescents with partly controlled (ORadj = 1.12, CI = 0.68- 1.83) and fully controlled (ORadj = 1.29, CI = 0.55- 3.01) asthma. | | | Asthma -> Lower Attainment | None | 12 | Satisfactory |
| REQUIA, W. J., SAENGER, C. C., CICERELLI, R. E., MONTEIRO DE ABREU, L. CRUVINEL, V. R. N. | Air quality around schools and school-level academic performance in Brazil. Atmospheric Environment, 279, | | | 2022 | | Country Wide | Brazil | | | Urban and rural | | Retrospective | | Cohort | | 7 | | We found that an increase of 10 μg/m3 in the long-term average PM2.5 around the Brazilian schools is associated with 2.98 points lower (95%CI: 0.37; 5.58) in the school-level academic performance (considering that the performance score varies from 0 to 1000). | | | Air Pollution -> Lower Attainment | None | 18 | High |
| RONCADA, C., DE OLIVEIRA, S. G.,  CIDADE, S. F.,  SARRIA, E. E.,  MATTIELLO, R.,  OJEDA, B. S.,  DOS SANTOS, B. R. L., GUSTAVO, A. D. S.,  PINTO, L. A.,  JONES, M. H.,  STEIN, R. T. &  PITREZ, P. M  . | Burden of asthma among inner-city children from Southern Brazil. The Journal of asthma: official journal of the Association for the Care of Asthma, 53, 498-504. | | | 2016 | | Porto Alegre | Brazil | | | Urban | | Contemporary | | Cross-section | | 1 | | School absenteeism (at least one day of missing school because of asthma) and sedentary behavior were high (57.1 and 67.2%, respectively). | | | Asthma -> Lower Wellbeing | None | 12 | Satisfactory |
| Authors | | Title | Year | | Region | | | | Country | | Environment | | Contemporary/Retrospective | | Cohort/ Cross-section | | Duration | | | Findings | Association Found | Use of linked health data | Quality Score | Overall Quality |
| SENTER, J. P., SMITH, B. M., PRICHETT, L. M.,  CONNOR, K. A. & JOHNSON, S. B. | | Pediatric Asthma Is Associated With Poorer 3-Year Academic Achievement in Urban Elementary and Middle-School Students. Academic pediatrics, 21, 1009-1017. | 2021 | | Northeast | | | | USA | | Urban | | Contemporary | | Cross-section | | 3 | | | Any asthma was associated with worse Measures of Academic Progress (MAP) performance across the 3 academic years. Students with the most significant asthma demonstrated worse performance on MAP and Partnership for Assessment of Readiness for College and Careers (PARCC). Aggregating across 3 school years, students scored 3.17points worse on MAP reading (95% confidence interval [CI]: 0.7−5.63; P= .012) and 3.56 points worse on MAP mathematics (95% CI: 0.52−6.6; P= .022); they had 48.8% (95%CI: 1.9% − 73.2%; P=.044) and 58.0%(95% CI: 21%−78%; P=.007) lower odds of proficiency on PARCC English/Language Arts and Mathematics, respectively compared to those without asthma. | Asthma -> Lower Attainment | None | 18 | High |
| SHENDELL, D. G., ALEXANDER, M. S., SANDERS, D. L.,  JEWETT, A. &  YANG, J. | | Assessing the potential influence of asthma on student attendance/absence in public elementary schools. The Journal of asthma: official journal of the Association for the Care of Asthma, 47, 465-472. | 2010 | | Georgia | | | | USA | | Urban | | Contemporary | | Cross-section | | 1 | | | Absence was higher (Average daily attendance (ADA) lower) among 4th grade asthmatic students compared to the entire classroom. | Asthma -> Absence | None | 14 | Satisfactory |
| SILVERSTEIN, M. D., MAIR, J. E., KATUSIC, S. K., WOLLAN, P. C., O'CONNELL, E. J. & YUNGINGER, J. W. | | School attendance and school performance: a population-based study of children with asthma. Journal of Pediatrics, 139, 278-283. | 2001 | | Minnesota | | | | USA | | Urban | | Contemporary | | Cross-section | | 36 | | | In the multivariable analysis of absenteeism, asthma (P < .006) and grade (P < .004), but not sex or decade, were significantly associated with absenteeism. | Asthma -> Absence | Yes | 18 | High |
| STRIDSMAN, C., DAHLBERG, E., ZANDRÉN, K. &  HEDMAN, L. | | Asthma in adolescence affects daily life and school attendance - Two cross-sectional population-based studies 10 years apart. Nursing open, 4, 143-148. | 2017 | | Northern region | | | | Sweden | | Not defined | | Retrospective | | Cross-section | | 2 | | | In 2013, the proportion reporting that asthma had an impact on daily life was higher among girls than boys (86% vs. 71%; test for trend p = .039). | Asthma -> Lower Wellbeing | None | 16 | High |
| STURDY, P., BREMNER, S., HARPER, G., MAYHEW, L.,  ELDRIDGE, S., EVERSLEY, J., SHEIKH, A., HUNTER, S., BOOMLA, K., FEDER, G., PRESCOTT, K. & GRIFFITHS, C. | | Impact of asthma on educational attainment in a socioeconomically deprived population: a study linking health education and social care datasets. PloS one, 7, e43977. | 2012 | | Tower Hamlets, London | | | | UK | | Urban | | Retrospective | | Cohort | | 3 | | | A weak positive association was found between overall school examination attainment and having active asthma (asthma treated with a bronchodilator during the past 12 months): b=0.066 (95% CI 0.013 to 0.119). No association was found for inactive asthma: b= 0.023 (95% CI -0.025 to 0.071). For these groups (active and inactive asthma) combined, a weak positive association was found: b=0.0495% CI (0.00to0.08). | Asthma! -> Lower Attainment | Yes | 19 | High |
| SULLIVAN, P. W., GHUSHCHYAN, V., NAVARATNAM, P., FRIEDMAN, H. S.,  KAVATI, A.,  ORTIZ, B. &  LANIER, B. | | The national burden of poorly controlled asthma school absence and parental work loss among school-aged children in the United States. Journal of Asthma, 55, 659-667. | 2018 | | Country Wide | | | | USA | | Not defined | | Retrospective | | Cohort | | 6 | | | School aged children (SAC) with asthma and an exacerbation had 1.8 times more missed school days per year than SAC without asthma. SAC with asthma but no reported exacerbation missed 1.3 times more school days than SAC without asthma. | Asthma -> Absence | None | 18 | High |
| SUNDBERG, R., TORÉN, K., HÖGLUND, D., ABERG, N. &  BRISMAN, J. | | Nasal symptoms are associated with school performance in adolescents. The Journal of adolescent health: official publication of the Society for Adolescent Medicine, 40, 581-583. | 2007 | | Gὂteberg Region | | | | Sweden | | Urban and rural | | Contemporary | | Cross-section | | 1 | | | The GLM analysis showed statistically significant associations between grade sum and both current rhinitis and severe nasal symptoms (SNS). | Asthma !-> Lower Attainment | None | 8 | Satisfactory |
| Authors | | Title | Year | | Region | | | | Country | | Environment | | Contemporary/Retrospective | | Cohort/ Cross-section | | Duration | | | Findings | Association Found | Use of linked health data | Quality Score | Overall Quality |
| ZHANG, T., WU, Y., GUO, Y., YAN, B., WEI, J., ZHANG, H., MENG, X., ZHANG, C., SUN, H. & HUANG, L. | | Risk of illness-related school absenteeism for elementary students with exposure to PM2.5 and O3. Science of the Total Environment, 842. | 2022 | | Jiangsu | | | | China | | Urban | | Contemporary | | Cohort | | 1 | | | An increase of 10 μg/m3 in the current-day concentration of PM2.5 and O3 was positively associated with illness-related absenteeism overall. The excess risk of absenteeism was 4.52 % (95% CI 4.37–4.67 %) for PM2.5 and 0.25 % (95% CI 0.01–0.36 %) for O3. The risk associated with O3 was boosted for the frequent absentees who tended to have basic diseases or were more vulnerable to infectious diseases. | Air pollution -> Absence | Yes | 18 | High |
| Aithal, S.S.,  Gill, S.,  Satia, I.,  Tyagi, S.K.,  Bolton, C.E. and  Kurmi, O.P. | | The effects of household air pollution (HAP) on lung function in children: a systematic review. International Journal of Environmental Research and Public Health, 18(22), p.11973. | 2021 | | N/A | | | | N/A | | Not defined | | N/A | | N/A | | N/A | | | Although the evidence suggests an inverse association between high exposure to HAP and lung function indices, there is a lack of longitudinal data describing this association. | N/A | N/A | 15 | Satisfactory |
| Al Ahad, M.A.,  Demšar, U.,  Sullivan, F. and  Kulu, H. | | Long term exposure to ambient air pollution and hospital admission burden in Scotland: 16 year prospective population cohort study. BMJ open, 14(12), p.e084032. | 2024 | | Scotland | | | | UK | | Urban and Rural | | Retrospective | | Cohort | | 16 | | | In fully adjusted models, the incidence rate for respiratory hospital admissions increased by 4.2% (95% CI 2.1% to 6.3%) (95% CI 0.8% to 1.7%) per 1 µg/m3 increase in PM2.5 and NO2 pollutants, respectively. | Air Pollution -> Asthma | Yes | 18 | High |
| Milojevic, A.,  Dutey-Magni, P.,  Dearden, L. and  Wilkinson, P. | | Lifelong exposure to air pollution and cognitive development in young children: the UK Millennium Cohort Study. Environmental Research Letters, 16(5), p.055023. | 2021 | | UK | | | | UK | | Urban and Rural | | Retrospective | | Cohort | | 7 | | | At age 5, particulate matter (PM2.5, PM10), nitrogen dioxide (NO2), sulphur dioxide (SO2) and carbon monoxide (CO) were associated with lower scores for Naming Vocabulary but no other outcome except for SO2 and Picture Similarity. | Air Pollution -> Lower Attainment | None | 18 | High |
| Sharma, R.,  Humphrey, J.L.,  Frueh, L.,  Kinnee, E.J.,  Sheffield, P.E. and Clougherty, J.E. | | Neighborhood violence and socioeconomic deprivation influence associations between acute air pollution and temperature on childhood asthma in New York city. Environmental research, 231, p.116235. | 2023 | | New York City | | | | USA | | Urban | | Retrospective | | Cohort | | 7 | | | We observed stronger main effects for PM 2.5 and SO 2 in the cold season on lag day 1 [4.90% (95% CI: 3.77–6.04) and 8.57% (5.99–11.21), respectively]; Tmin in the cold season on lag day 0 [2.26% (1.25–3.28)]; and NO 2 and O 3 in the warm season on lag days 1 [7.86% (6.66–9.07)] and 2 [4.75% (3.53–5.97)], respectively. Violence and SDI modified the main effects in a non-linear manner; contrary to hypotheses, we found stronger associations in lower-violence and -deprivation quintiles. At very high stressor exposures, although asthma exacerbations were highly prevalent, pollution effects were less apparent—suggesting potential saturation effects in socio-environmental synergism. | Air Pollution -> Asthma | None | 18 | High |
